# Investigation of Age-Associated Cognitive Functional Homophily in Community-Dwelling Older Adults’ Confidant Social Networks Using Exponential Random Graph Model

**DOI:** 10.3390/ijerph19084574

**Published:** 2022-04-11

**Authors:** Ayako Morita, Yoshimitsu Takahashi, Takeo Fujiwara

**Affiliations:** 1 Department of Global Health Promotion, Tokyo Medical and Dental University, Tokyo 153-8510, Japan; fujiwara.hlth@tmd.ac.jp; 2 Department of Health Informatics, Kyoto University School of Public Health, Kyoto 606-8317, Japan; takahashi.yoshimitsu.3m@kyoto-u.ac.jp

**Keywords:** network analysis, homophily, age-associated cognitive function, old, Japan

## Abstract

One of the prominent interventions to tackle loneliness and social isolation in older adults is social facilitation. The present study investigated whether similarities in cognitive functions that are sensitive to age play a role in confidant social networks among older adults. We analyzed the data of 252 community-dwelling older adults in Wakuya City, Miyagi Prefecture, Japan, who responded to a self-administered questionnaire and cognitive health checkups provided by the city in 2017. We performed Exponential Random Graph Model and investigated educational attainment, orientation, word registration, clock drawing, delayed recall, verbal fluency and logical memory homophily while adjusting for density, reciprocity, age, sex living arrangement, presence of disability in instrumental activities of daily living, educational attainment and cognitive impairment status. The probability of a confidant tie with an older adult was significantly reduced by 6% (odds ratio (OR): 0.94, 95% confidence interval (CI): 0.90–0.99) for one score difference in logical memory, and marginally increased by 5% (OR: 1.05; 95% CI: 1.00–1.11) for one score difference in delayed recall. There was no significant association between educational attainment and other age-associated cognitive functional scores. Our findings suggest that similar logical memory functions play a role in strong social network building among community-dwelling older adults in Japan.

## 1. Introduction

Loneliness and social isolation in older adults are major public health concerns. They refer to functional and structural aspects of poor social relations [1,2], and their estimated prevalence is as high as 30% for loneliness and 55% for social isolation [3,4]. Many older adults experience the loss of one or more close relationships due to age-associated life events (e.g., losses of spouse, relatives or friends, losses of social roles, declined health and functional capacity) [5]. A lack of people whom they can talk to or count on in times of trouble imposes high stress, poor coping and serious health consequences on older adults. There is strong evidence that lonely, socially isolated older adults are at elevated risk of maladaptive health behaviors, serious medical conditions such as cardiovascular disease, depression, suicide and mortality from all causes [6].

One of the prominent interventions to tackle loneliness and social isolation in older adults is social facilitation—bridging people and facilitating them to build meaningful relations [7,8]. Network studies report “homophily” (i.e., the tendency where people who are similar are more likely to be associated) is frequently observed in empirical settings and proposed as a strong driving force for the initiation and maintenance of social relationships [9]. The population of older adults is not a homogenous one but characterized by cognitive variance in fluid intelligence, which is enlarged in the normal course of aging and progression of age-associated neurodegenerative diseases [10]. Cognitive homophily on the basis of general intelligence, academic achievements and educational attainment was reported among high school friends, college roommates and spouses, including a few older adult participants [11,12,13,14,15,16,17]. Similarities in age-associated cognitive function may be a key driving force of strong social ties in older adults; however, no study has investigated this hypothesis to date.

In an examination of homophily, it is important to rule out the possible structural effect because social relations are not purely independent but built upon social structure instead [18]. It is also important to examine adjusting for individual attribute effects because individual differences in cognitive function may affect the likelihood of forming confidant relations (i.e., older adults with higher cognitive function may be more popular and those with higher cognitive function are likely to form confidant relations with others with higher cognitive function) [18]. The Exponential Random Graphic Model (ERGM) analysis allows the investigation of homophily effects in the observed whole network while controlling for both structural and individual attribute effects [19].

Japan is the world’s most super-aged nation, with almost one-third of the population being 65 years and above as of 2020 [20], and one in every six of them suffer from dementia [21]. Moreover, the country has also been named as one of the loneliest or socially isolated countries in the world (e.g., 20% of older adults living alone, and 25.9% of seniors aged 60+ do not have any close friends outside their family compared to 11.9% in the United States, 17.1% in Germany and 8.9% in Sweden as of 2015) [4,20]. In 2021, the government appointed a cabinet minister in charge of loneliness and social isolation to countermeasure the current situation. The aim of the present study was to examine whether similarities in age-associated cognitive function play a significant role in confidant social networks among older adults using the ERGM analysis.

## 2. Materials and Methods

### 2.1. Participants

A self-reported survey was distributed in late August 2017 to all the residents affiliated with the national health insurance or late-stage medical care system and turning 65 years or older during the Japanese fiscal year of 2017 in Wakuya City, Miyagi Prefecture, Japan (*n* = 4902). These two public health insurance cover 87.3% of residents aged 65 and above as of 2017 [22]. In total, 1722 agreed to participate and returned the completed questionnaire (response rate: 35.1%) by mail. A total of 906 responses were eligible for the social network analysis, and the current study analyzed 252 responses that were able to tie with cognitive health checkups data of the same year. Cognitive health checkups were provided to community-dwelling residents aged 65+ as part of the national health insurance and late-stage medical care system, and none of the participants were diagnosed with dementia or under medication relevant to the treatment of dementia.

### 2.2. Aging-Related Cognitive Function

Age-associated cognitive function was assessed by the Japanese version of the Quick Mild Cognitive Impairment Screen (Qmci-J), which showed the good concurrent validity against the standardized Mini-Mental State Examination (sMMSE) and moderate positive correlation with it with a very similar cut-off to the original version in community-dwelling Japanese older adults [23]. Qmci is a reliable and valid screening tool for cognitive impairment based on the assessment of orientation (awareness of time and place), word registration (immediate recall of five words), clock drawing (drawing a clock face with a specified time), delayed recall (recall of the five words two and half a minute after), verbal fluency (a total number of words relating to a named category), and logical memory (immediate verbal recall of a short story). Each domain score ranged from 0 to 10, 0 to 5, 0 to 15, 0 to 20 and 0 to 30, respectively, and a total score ranged from 0 (indicating serious cognitive impairment) to 100 (no indication of cognitive impairment) [24].

### 2.3. Confidant Social Network

We measured whether or not and with whom participants formed confidant ties. In order to identify confidants within the household, we asked the participant to list their household members. We identified the spouse as a confidant if the participant rated marital satisfaction as six points or higher on a 10-point Likert scale or rated five points or lower but reported the presence of someone to complain to and/or consult problems with. With respect to non-co-residing confidants, we asked the participants to provide “real name”, “sex”, “residential address up to district name” and “age” of whom they disclosed their worries and anxiety in their community. After excluding those living outside of Wakuya City, we identified the same person with the following criteria: (1) their first and family names were spelled in the same Japanese kanji or hiragana), (2) their sex were matched, (3) their age difference was less than three years and (4) their residential districts were matched.

### 2.4. Demographics

We obtained information about age, sex and educational attainment (i.e., elementary school equivalence or less, junior high school equivalence, high school equivalence or college and above) from the cognitive health checkups data and higher-level functional independence based on the Tokyo Metropolitan Institute of Gerontology Index of Competence (TMIG-IC) by the questionnaire. As for education, we classified the participants with elementary school equivalence or less and junior high school equivalence in one category since there were few who received formal education of equivalent or less than elementary school. As for functional independence, we applied 12/13 cut-off scores [25].

### 2.5. Statistical Analysis

We assumed there was a tie (i.e., social connection) between two nodes (i.e., participants) when at least one of them named the other as a confidant. The network density was computed by the observed number of ties divided by the total number of potential ties among the participants, which is “n” multiplied by “n-1” divided by two. We performed ERGM analysis using Markov Chain Monte Carlo (MCMC simulation) on our directed network, using the ergm package on R version 4.0.3. Following the literature and text [26,27], we specified *Y_ij_* as the probability of a confidant dyadic tie between the node i and the node j being present (1) or absent (0) and examined the probability of a tie being present (1) as two nodes differ by one educational attainment category and one score on each Qmci-J sub-scale scores (i.e., orientation, registration, clock drawing, delayed recall, verbal fluency and logical memory). Then we calculated the coefficient of coefficients-the log-odds likelihood of any confidant tie with another older adult and converted the log-odds to compute odds ratio (OR) using the following formula: exponential (edge coefficient)/{1 + exp (edge coefficient)}. The 95% confidence interval (CI) was computed by exponential (edge coefficient ± 1.96 ×standard error)/{1 + exponential (edge coefficient ± 1.96 × standard error)}. Adjusting the effects of edges (i.e., a total number of ties), reciprocity (i.e., the tendency that if A says that she nominates B as a confidant, B is more likely to nominate A as a confidant, irrespective of other factors) and individual actors’ demographics and cognitive impairment status, decreased OR for a tie formation by the increased cognitive functional difference between nodes indicates cognitive homophily (i.e., the tendency that A is more likely to nominate B as a confidant if A and B have similar cognitive functions), and increased OR indicates cognitive heterophily (i.e., the tendency that A is less likely to nominate B as a confidant if A and B have similar cognitive functions). The Akaike information criterion (AIC) and the Bayesian information criterion (BIC) were computed to check the model fit.

## 3. Results

Table 1 summarizes the participants’ characteristics. Our participants were 73.4 (SD = 6.1) years old on average, and slightly more than half of them were female. The most common educational attainment was high school (58.7%), followed by junior high school or less (23.8%). About 40% of the participants had a higher-level functional disability, and 46.7% were suspected of cognitive impairment (the average total score of the Qmci was 58.9 ± 12.0, and the average sub-test score was 9.7 ± 0.8 for orientation, 3.8 ± 1.1 for word registration, 13.7 ± 2.9 for clock drawing, 15.1 ± 5.2 for delayed recall, 7.1 ± 2.4 for verbal fluency and 9.5 ± 5.9 for logical memory). Of the 252 participants, 83.7% (*n* = 211) were living with another person (young/old), and 73.0% (*n* = 184) had at least one confidant (young/old) outside the household but within the same city. After excluding unsatisfied spousal ties (*n* = 8), we identified 79 ties, with 54 ties being two-way directed. Among them, 35 ties were directed to friends, relatives or neighbors outside the household.

Table 2 presents the results of the ERGM analysis. In the final model (AIC = 847; BIC = 1019), we observed a significant inverse association between differences in logical memory scores and a formation of confidant relational tie (coefficient = −0.057, SE = 0.026, *p* = 0.029). One score difference in logical memory decreased the probability of confidant tie formation (OR: 0.94, 95% Confidence Interval: 0.90, 0.99), indicating people who are similar in logical memory scores were more likely to form the tie. On the other hand, we observed a tendency of a positive association between absolute differences in delayed recall scores and a formation of confidant relational tie (coefficient = 0.050, SE = 0.028, *p* < 0.10) with OR of 1.05 (95% Confidence Interval: 1.00, 1.11). Neither significant homophily nor heterophily effects were observed with respect to educational attainment and cognitive functions in orientation, registration, clock drawing and verbal fluency.

**Figure 1:**
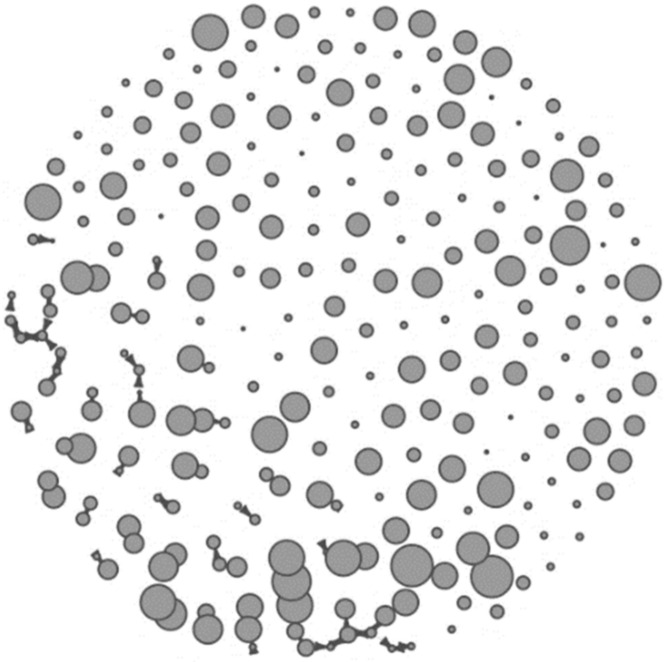
Visual representation of confidant network.

Figure 1 presents the visual association of logical memory scores with the confidant network in our participants. While the score difference between the connected nodes ranged from 0 to 22, 15 ties (19%) were established between the nodes of the same size (=the same logical memory score). More than half (55.7%) of the ties were established between the nodes with less than five score differences, and the ties with more than ten score differences accounted for less than 10% (*n* = 6).

Nodes represent participants, and ties between nodes represent one or two-way confidant relational ties, where an arrow indicates a direction. Nodes without any tie were isolates. Node size corresponds to the participants’ logical memory score (larger nodes reflect better function).

## 4. Discussion

This is the first study to investigate the age-associated cognitive functional homophily in the confidant social network of community-dwelling older adults using ERGM analysis. Having adjusted for several structural and individual attributable factors, we observed dissimilarity in logical memory function to be associated with a decreased probability and similarity in delayed recall function to be associated with a higher probability to form a confidant tie with another older adult in the same community. On the other hand, we document neither significant homophily in educational attainment nor other age-associated cognitive domains (i.e., orientation, registration, clock drawing and verbal fluency).

Connecting to others who are similar is a fundamental building block of social relations. Our documentation of logical memory homophily is consistent with earlier network studies on high school friends, college roommates and marital partners that documented cognitive homophily [11,12,13,14,15,16,17]. However, confidant social networks of community-dwelling older adults were not based on educational attainment as reported previously in young populations but based on logical memory. Logical memory is one of the most salient cognitive domains that degenerates with age and is most sensitive to detecting cognitive impairment among all the Qmci measurements [24]. Consistent with our findings, a study in the U.S. reported that health behavioral characteristics of older adults’ egocentric social networks were significantly positively associated with health behaviors (i.e., health behavioral homophily), but educational attainment was not a significant predictor [28]. Older adults may estimate whether they have a similar cognitive ability to themselves on the basis of logical memory during communication rather than educational attainment a few to several decades ago.

We also found marginally delayed recall heterophily on confidant tie formation, which was contrary to the hypothesis. However, it is common for older adults to have non-reciprocal social support from younger generations who have better cognitive functions [9]. The tip of the tongue, temporary inability to retrieve familiar words and names, is the most commonly reported memory error that older adults experience in the normal course of the day [29]. Experiences of receiving and/or providing help with common memory errors may serve to build a confidant relationship with other older adults rather than discouraging it.

Although our data and findings are unique, we must note several limitations of the study. First, our study participants were residents of a small city in rural Japan and did not compose of older adults who were affiliated with the social insurance system and those who chose not to participate in the survey/cognitive health checkups. Sharing similar social backgrounds and health statuses could lead us to over-estimate cognitive homophily, although we documented a variation in age-associated cognitive functions, particularly with respect to delayed recall and logical memory, among our participants. Still, the generalizability of our findings in a bigger urban community and different countries remains an important research topic for future studies. Second, some of our participants were cognitively impaired. Dementia status could affect the actual number and the recalled number of confidant relational ties [30,31], which would lead to overestimating homophily effects. However, none of the participants were medically diagnosed with dementia and managed to live independently in the community. Furthermore, we adjusted for the effects of cognitive impairment on a probability of a confidant tie formation by adding individual actors’ cognitive impairment status in the model when we investigated homophily effects. Third, we did not investigate physical and mental health homophily. Investigation of other health attributes would help us to understand the nature of confidant social networks in later life. Finally, Framingham social network studies indicated several health behaviors as risk factors for cognitive impairment that is spread from person to person integrated into the same social network [32,33,34]. However, our data were cross-sectional, and thus, we cannot infer whether age-associated cognitive homophily was a result of selection or influence, or both.

The number of older adults living in single-person households has been increasing, and one in every four Japanese seniors lack confidants other than their families [4,20]. Japan has 89,498 senior social clubs [35] and 5249 long-term care preventive care facilities and service providers [36]. In order to promote confidant tie formation among community-dwelling older adults, facilitators may consider putting people who are similar in the ability to process and recall conversations in the same group rather than grouping them at random in activities. Moreover, we should pay attention to the activity contents. In addressing their failed peer support telephone dyad intervention, Heller and his colleagues advocated that activities in which participants share events that require coping “currently” would be essential in facilitating new “confidant” relation-building [37]. Providing pair or group-based talk opportunities on current problems among community-dwelling older adults with a similar level of logical memory may therefore be useful in building social networks where the community-dwelling older adults consult each other on their daily problems and challenges. On the other hand, some community-dwelling older adults may not fit in or drop out of the group due to the progression of age-associated cognitive impairment. An alternative strategy for social facilitation in such cases is befriending, where clinicians, care providers or social workers connect with volunteers who would be willing to provide social support and companionship on a regular basis [7,8].

## 5. Conclusions

The similarity in logical memory and dissimilarity in delayed recall functions, but not educational attainment, was associated with an increased probability of the confidant social network among the community-dwelling older adults in Japan.

## Figures and Tables

**Table 1:** Demographic and age-associated cognitive functional characteristics of the participants (*n* = 252).

Variables	*n*	%	Mean (SD)
Age	252		73.4 (6.1)
Sex			
Male	113	44.8	
Female	139	55.2	
Living arrangement (alone)			
Alone	41	16.3	
With other	211	83.7	
Educational attainment			
Junior high school or less	60	23.8	
High school	148	58.7	
College and above	44	17.5	
Higher-level functional capacity			
No impairment	139	55.2	
Disability present	99	39.3	
Missing	14	5.6	
Cognitive impairment			
Likely normal	136	54.0	
Suspected mild cognitive impairment	73	29.0	
Suspected dementia	43	17.1	

**Table 2:** Exponential Random Graph Models predicting confidant ties among older adults based on difference in age-associated cognitive functions between nodes among the participants (*n* = 252).

Variables	Adjusted OR	95% CI	*p*-Value
Difference in educational attainment			
Same level	1.00		
One level of difference	0.68	(0.35, 1.29)	0.24
Two levels of difference	0.39	(0.10, 1.47)	0.17
Difference in age-associated cognitive function			
One score difference on orientation	1.14	(0.85, 1.53)	0.37
One score difference on registration	0.90	(0.68, 1.18)	0.41
One score difference on clock drawing	1.01	(0.94, 1.08)	0.83
One score difference on delayed recall for words	1.05	(1.00, 1.11)	0.075
One score difference on verbal fluency	1.02	(0.90, 1.15)	0.76
One score difference on logical memory	0.94	(0.90, 0.99)	0.029
Akaike Information Criterion = 847			
Bayesian Information Criterion = 1019			

Notes: adjusted OR = odds ratio in the model adjusted for the effects of edges, reciprocity and individual actors’ sex, age, living arrangement, higher-level functionality, educational attainment and level of cognitive impairment in a probability of a confidant tie formation.

## Data Availability

The datasets generated during and/or analyzed during the current study are available from the corresponding author upon reasonable request and permission from the Institutional Review Board.
